# Change in grip strength, hang time, and knot tying speed after 24 hours of endurance rock climbing

**DOI:** 10.3389/fspor.2023.1224581

**Published:** 2023-08-01

**Authors:** Elaine Yu, Jacques Lowe, Jasmin Millon, Kristi Tran, Christanne Coffey

**Affiliations:** ^1^ Department of Emergency Medicine, University of California San Diego, San Diego, CA, United States; ^2^ Carle Illinois College of Medicine, University of Illinois Urbana-Champagne, Urbana, IL, United States; ^3^ American University of the Caribbean School of Medicine, Cupecoy, Sint Maarten; ^4^ Department of Emergency Medicine, State University of New York Downstate, Brooklyn, NY, United States

**Keywords:** rock climbing, endurance, grip strength, hang time, speed

## Abstract

**Background:**

Non-professional climbers are increasingly attempting long routes in a single day. Many suffer injury or rely on search and rescue teams when they become too fatigued to finish. Predicting fatigue is difficult, and existing studies have only studied climbers over durations less than an hour, while many outdoor multipitch climbs require more than an hour of climbing.

**Objectives:**

To determine how strength, endurance, and dexterity reflect fatigue after 24 h of continuous climbing.

**Methods:**

Volunteer competitors completed measurements of grip strength, static hang time to failure, and time to tie a figure-eight follow-through knot. Measurements were taken during the registration period before the competition and again within an hour after the competition ended. Measurements were compared using the paired *t*-test. Subgroup analysis was applied to competitors by division. Linear regression was applied to determine the relationship between vertical feet climbed and the number of routes climbed during the competition on each metric.

**Results:**

Thirty-six total climbers (average age 29.4 years old) completed pre- and post-competition measurements. After 24 h of climbing (*n* = 36), mean grip strength decreased by 14.3–15 lbs or 14.7%–15.1% (*p* < 0.001) and static hang time decreased by 54.2 s or 71.2% (*p* < 0.001). There was no significant change in time to tie a figure-eight-follow-through knot. Grip strength and hang time decreases were significant in climbers with outdoor redpoints of 5.10a and above. Hang time decreased by 5.4 s per 1,000 vertical feet climbed (*p* = 0.044).

**Conclusion:**

Climbers can expect to experience a 14.7%–15.1% decrease in grip strength and 71.2% decrease in static hang time after 24 h of continuous climbing. These changes may make it difficult to climb consistently over a long objective, and climbers can use these measures at home to train for longer climbing routes. Future studies on shorter climbing intervals can help determine rates of decline in performance measures.

## Introduction

The sport of rock climbing has experienced a rapid increase in participation over the past several years. The sport's increasing popularity is evidenced by increasing numbers of climbing gyms opening all over the USA and Climbing officially becoming an Olympic sport for the 2020 Tokyo games (1–3). In 2019, industry leaders released the first ever State of Climbing report which noted a yearly increase in 440,000 climbers (4). Paralleling this growth, the US has also experienced a dramatic increase in climbing-related injuries seen in emergency departments from 1990 to 2016 (5, 6).

The American Alpine Club, which publishes an annual report of climbing accidents, notes an increase in accidents from its first publication in 15 in 1951, surpassing 100 in 1970, exceeding 200 in 1986, and staying steadily above 100 since 1982 (7, 8). Between 1951 and 2016, exceeding abilities and/or inexperience was the second most common direct cause of accidents after falls (7). Of all the indirect causes leading to accidents from 1951 to 2020, inexperience was second only to climbing without a rope (8).

There is a growing concern about climbing's overall safety for newer climbers who venture outdoors. Epidemiological literature describes that the incidence of climbing injuries is greater in outdoor climbing on real rock than that of indoor climbing in a gym (9). Furthermore, there has been a documented increase in search and rescue efforts nationwide within the National Park Service (10), and while limited, rescue efforts for climbing-related incidents have also been of notable interest in both the US and Europe (11, 12). Inexperience and fatigue have been cited as increasing contributors to these reported search and rescue efforts (13, 14).

As more climbers venture outdoors for recreational sport and traditional climbing, it is important to recognize the physical limitations athletes experience on routes that may be far longer than found in gyms, and when managing weather and other environmental factors not replicable in gym settings. Newer climbers, or those who may climb infrequently, may be at increased risk of injury due to their inexperience or lack of physical conditioning (15). Several studies have been performed in other realms of endurance sports such as marathon running to better understand factors such as age (16) and gender (17) as they relate to overall performance. While there are articles that explore physiologic characteristics (18, 19) that may be associated with increased climbing performance, there remains a paucity of literature that describes factors related to climbers who undergo continuous climbing feats (20).

Grip strength has been shown to decrease by 22.1%–23% after a 30-minute bout of indoor sport rock climbing (21), grip strength recovers within 10 min after a 2-minute bout of indoor climbing (22), and maximum hang time has been shown to decrease over eight repeated hangs with 1-minute rest intervals and plateau with 3-minute rest intervals (23). Outdoor rock climbing on long single-day routes often requires more than 30 min of climbing. Due to the nature of different routes, climbers may be required to hang with their entire body weight with rest intervals much shorter or longer than 1–3 min. Additionally, multipitch climbing with a partner requires multiple changeovers between belaying and climbing, necessitating finger dexterity in knot tying, clipping, and belaying.

Due to the limited available data on endurance climbing and how climbers' performance may be affected by multiple hours of continuous rock climbing, we sought to determine how strength, endurance, and dexterity reflect fatigue after 24 h of continuous climbing. We measured grip strength, hang time, and knot-tying time, as these are easily replicated measurements that do not require training or access to expensive or heavy equipment. Our secondary objectives were to determine how climbing ability, vertical distance climbed, and the total number of routes climbed affected those performance metrics.

## Materials and methods

### Setting

Measurements were taken during the 24 h of Horseshoe Hell event hosted at Horseshoe Canyon Ranch in Jasper, AR from September 23 to 24, 2022. Pre-event measurements taken on September 23rd between 7 and 9 AM; post-event measurements were taken on September 24th between 10 and 11 AM. The weather during the event was partly cloudy with temperatures ranging between 64 and 88 F, winds 0–10 mph, and humidity between 37% and 71%. There was no precipitation during the event.

The rock at Horseshoe Canyon Ranch is primarily limestone. Routes range in height from 30 to 90 ft and in difficulty from 5.2 to 5.14 (Yosemite Decimal System) in either sport, traditional, or mixed styles, which is equivalent to Lower Grade through Higher Elite in the IRCRA system (24). Each route is given a point value based on a combination of its height, difficulty, and style. Climbers are required to complete each route “clean” by free climbing and not weighting the rope to claim points for the ascent; free-soloing is expressly prohibited.

Climbers compete in pairs, swapping between belaying and climbing so that both can log points for the competition. While some climbers choose to climb different routes, most partner pairs climb the same routes and complete routes in sequence spanning an entire cliff face so that they are only traveling several feet between finishing one route and starting the next, with longer breaks in climbing to travel between cliff faces. Each team's time management in the 24 h must factor in route finding, waiting in line for other teams to complete routes, eating, drinking, toileting, traveling between routes and climbing areas, and attempting more difficult climbs during daylight hours. Pairs are self-supported and must carry their own gear, food, water, and other necessities with them as they climb throughout the ranch.

Completed routes are recorded via the honor system on a phone application or physical scorecard. Scorecards are posted on the event website and results are publicly available. We referenced the total vertical feet climbed, number of routes climbed, and entry division from scorecards.

### Participants

Study participants were recruited during the registration check-in period before each competition. We collected their age, sex, gender identity, and competition category. In total, 54 competitors agreed to participate. After signing an informed consent, participants were assigned a random number and given a wristband with that number to de-identify them for testing. Their wristband number was documented on their informed consent and kept in a secured file folder by one research assistant. Spectators and support personnel were excluded. Participants voluntarily completed the study measurements at the time of enrolling in the study and again within 1 h of finishing each competition.

Each climber entered the competition in the division based on their highest outdoor redpoint: 5.9 for Recreational, 5.10d for Intermediate, 5.12a for Advanced, and 5.12b+ for Elite. These divisions are set by the competition organizers and are the same for both male and female entrants. Per the IRCRA Reporting scale, these categories would correlate with Lower Grade, Intermediate, Advanced (Female), and Advanced (Male)/Elite/Higher Elite (24). Entry into the competition is via a lottery system or by climbing a minimum number of routes in the prior year of the same competition. While professional climbers have competed in the past, most competitors are recreational climbers.

### Measurements

We measured three parameters during this study: grip strength, hang time, and knot-tying time. These were chosen because they were tests that could be reproduced by average climbers not requiring any special athletic training and with equipment that could be easily purchased and transportable by both traveling climbers and the research team to the event location.

### Protocol

Participants completed these exercises in a randomized order before and another randomized order after each competition. All measurements were recorded on paper next to the wristband number of the participant to protect identifying information. Each participant was given one attempt per measurement. Maximum hand grip strength (in pounds, Handeful handheld dynamometer) was measured in for both the right and left hands of each participant in their preferred arm positioning while standing. Most participants chose to do so in a slightly bent arm position where they could see the numbers on the screen changing as they gripped down. The maximum number stays displayed on the screen once the hand lets go.

**Figure 1:**
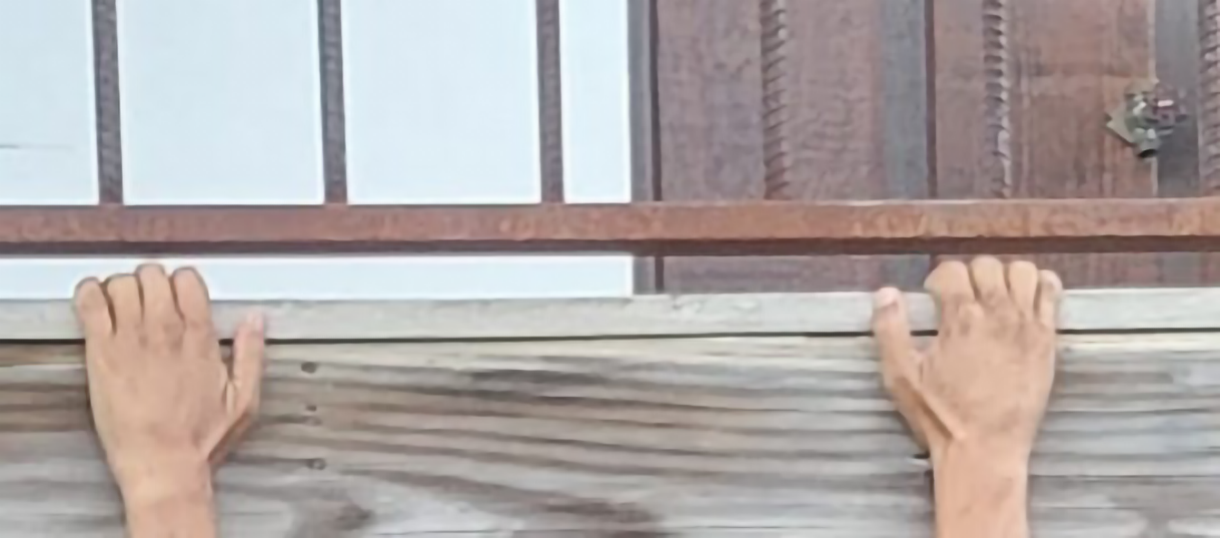
Hand positioning during hang time test, photo taken during live data collection by Elaine Yu.

Hang time was measured by having participants hang until failure from the edge of an elevated porch balcony that is constructed out of wooden planks with a 90-degree angle at the edge, a depth of approximately six feet, and a length of approximately 50 feet. Their feet were not allowed to touch the ground while hanging, but participants could select their preferred distance between their hands and position their body however they preferred if their feet did not touch the ground. Most participants chose to hang with their proximal interphalangeal joints flexed and metacarpophalangeal joints extended (see Figure 1). Two matching commercially available hanging grips were initially selected for this measurement, however there was no place to hang them close to the registration area, therefore the porch edge immediately behind the registration tent was used instead.

For knot-tying time, each participant wore an adjustable 8 mm cordelette around their waist at their preferred tightness to simulate a tie-in point on a climbing harness. The cordelette was used in lieu of each participant's personal climbing harness because none had their harness available at the time of registration. Participants were timed on their speed in tying a figure-8-follow-through knot with a provided length of 9.7 mm dynamic climbing rope through this improvised harness, starting when a research assistant called “start” and started a stopwatch, and ending when the participant called “done” and the stopwatch was stopped.

## Results

### Participants

**Figure 2:**
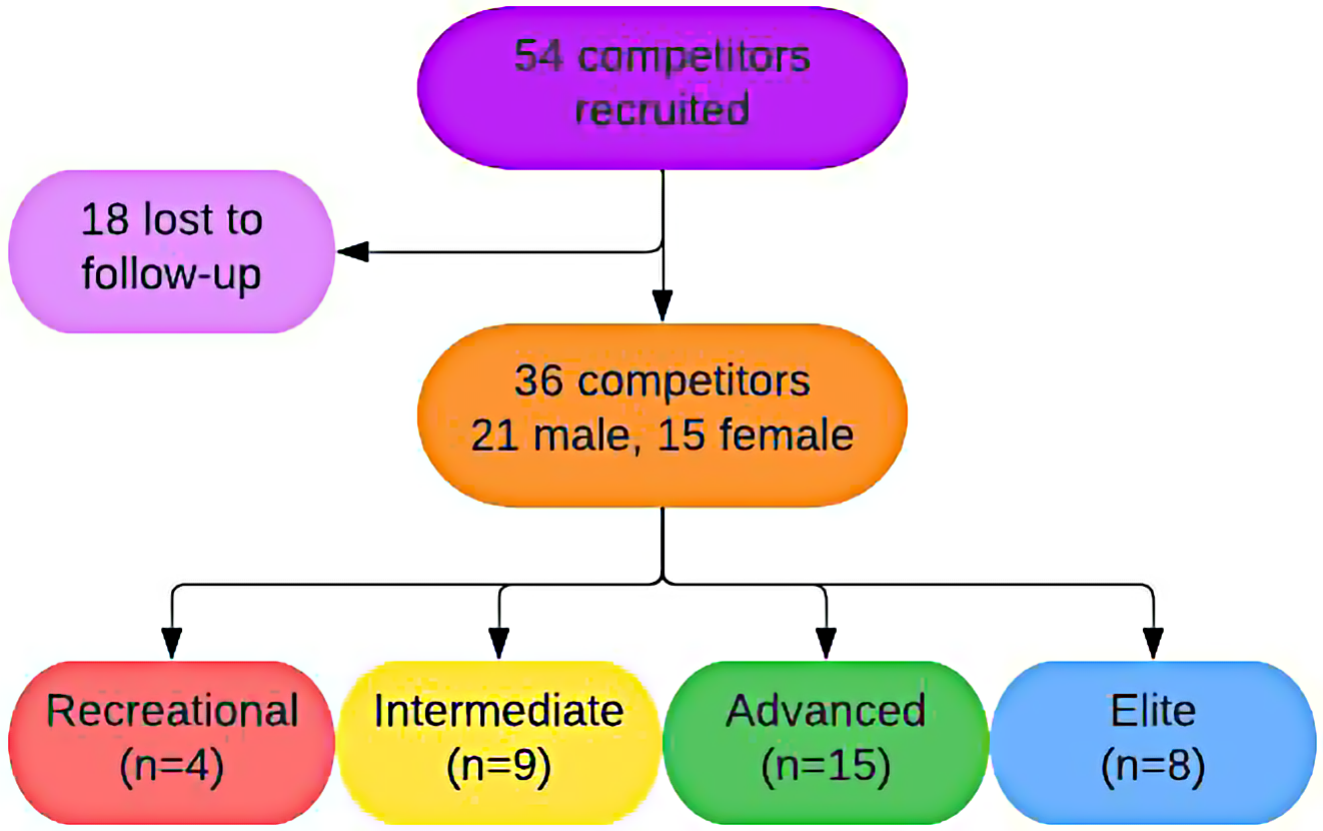
Participant characteristics. Created by Elaine Yu.

Thirty-six competitors completed repeat measurements. There were four competitors in the Recreational category, nine competitors in the Intermediate category, 15 competitors in the Advanced category, and eight competitors in the Elite category. Climbers ranged from 21 to 40 years of age, with an average age of 29.4 years old; 21 were male, 15 were female. See Figure 2. In some cases, measurement recordings were either missing or illegible and unable to be included in calculations. Any pair of measurements that was recorded both pre- and post-competition for an individual was included in the calculations.

### Statistical analysis

Percent decreases in handgrip strength from Macdonald et al. were used to determine a sample size of 8 with an alpha level of 0.5 and a power of 0.8.

We applied the paired two-tailed t-test to determine the mean difference between the pre- and post-competition data. Additionally, we performed the paired two-tailed *t*-test on subgroups based on their entry division. The *α* for each paired test was adjusted by Bonferroni correction with the resulting *α*_adjusted_ value used to determine significance. Cohen's *d* effect sizes were used. Percent increase or decrease in value was also calculated. We applied linear regression to predict the change in grip strength, hang time, and knot-tying time based on vertical height climbed and the number of routes climbed. *p* < 0.05 was used to determine significance.

**Figure 3:**
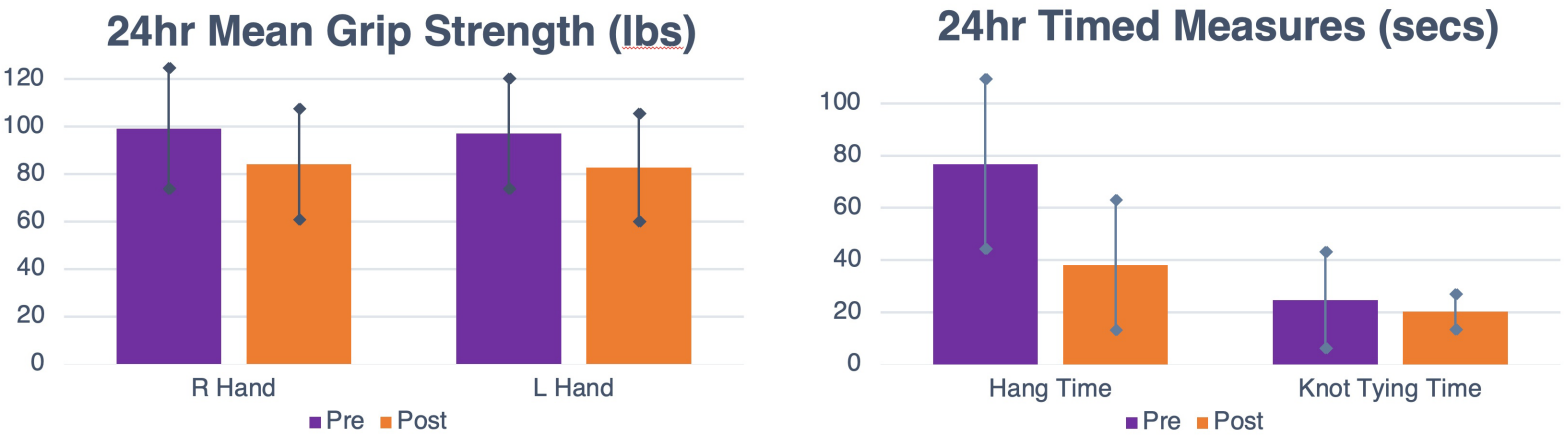
Left: Pre- and post-competition grip strength measurements in pounds of the right and left hands. Right: Pre- and post-competition hang time and knot tying time measurements in seconds.

In the 24-hour competition, the mean right hand grip strength before the competition was 99.2 lbs (SD = 25.5) and after the competition was 84.2 lbs (SD = 23.3); [*t*(34) = 6; *p* < 0.001; *d* = 1.01; *α*_adjusted _= 0.001]; or a 15.1% decrease. Mean left hand grip strength before the competition was 97 lbs (SD = 23.3) and after the competition was 82.7 lbs (SD = 22.7); [*t*(31) = 8; *p* < 0.001; *d* = 1.41; *α*_adjusted _= 0.002]; or a 14.7% decrease. Mean hang time before the competition was 76.8 s (SD = 32.6) and after the competition was 38.1 s (SD = 24.9); [*t*(34) = 12.4; *p* < 0.001; *d* = 2.1; *α*_adjusted _= 0.001]; or a 71.2% decrease. Mean knot tying time before the competition was 24.7 s (SD = 18.5) and after the competition was 20.2 s (SD = 6.8); [*t*(28) = 1.3; *p* = 0.218; *d* = 0.23; *α*_adjusted _= 0.002]; or a 18.2% decrease. Changes are displayed in Figure 3.

On subgroup analysis, there was a significant change in hang time in all competition categories. Grip strength of both the right and left hands showed a significant change in Intermediate, Advanced, and Elite climbers, but not Recreational climbers. Change in knot tying time was not significant in any category. Subgroup analysis by division are presented in Table 1.

**Table 1:** Subgroup analysis by division.

	Right hand grip strength	Left hand grip strength	Hang time	Knot tying time
Recreational (highest outdoor redpoint 5.9)				
Before	*M* = 74.3 lbs	*M* = 76.5 lbs	*M* = 43.7 s	*M* = 39.4 s
	SD = 13	SD = 9	SD = 11.8	SD = 24.8
After	*M* = 72.9 lbs	*M* = 69.5 lbs	*M* = 15.3 s	*M* = 27.2 s
	SD = 8.7	SD = 10.3	SD = 15.4	SD = 13.5
Change	Δ = 1.4 lbs, −1.9%	Δ = 7 lbs, −9.2%	Δ = 28.4 s*, −65%	Δ = 12.2 s, −31%
	*t*(3) = 0.3	*t*(3) = 2.2	*t*(3) = 9.7	*t*(3) = 0.9
	*p* = 0.757	*p* = 0.116	*p* = 0.002	*p* = 0.448
	*α*_adjusted_ = 0.013	*α*_adjusted_ = 0.013	*α*_adjusted_ = 0.013	*α*_adjusted_ = 0.013
Intermediate (highest outdoor redpoint 5.10d)				
Before	*M* = 112.7 lbs	*M* = 106.8 lbs	*M* = 64.9 s	*M* = 22.5 s
	SD = 32.4	SD = 29.5	SD = 14	SD = 11.8
After	*M* = 90.1 lbs	*M* = 89.5 lbs	*M* = 22.1 s	*M* = 20.3 s
	SD = 16.3	SD = 22.1	SD = 20.9	SD = 4.3
Change	Δ = 22.6 lbs, −20.1%	Δ = 17.3 lbs, −16.2%	Δ = 47.3 s*, −65.9%	Δ = 2.2 s, −9.8%
	*t*(8) = 2.9	*t*(6) = 3.2	*t*(8) = 7	*t*(7) = 0.4
	*p* = 0.019	*p* = 0.019	*p* < 0.001	*p* = 0.708
	*α*_adjusted_ = 0.006	*α*_adjusted_ = 0.007	*α*_adjusted_ = 0.006	*α*_adjusted_ = 0.007
Advanced (highest outdoor redpoint 5.12a)				
Before	*M* = 94.2 lbs	*M* = 95.1 lbs	*M* = 89.3 s	*M* = 25.1 s
	SD = 22.6	SD = 22.8	SD = 42	SD = 22.5
After	*M* = 80.4 lbs	*M* = 81.3 lbs	*M* = 28.2 s	*M* = 20.1 s
	SD = 30.3	SD = 26.5	SD = 32.6	SD = 5.9
Change	Δ = 13.8 lbs*, −14.6%	Δ = 13.8 lbs*, −14.5%	Δ = 61.1 s*, −68.4%	Δ = 5 s, −19.9%
	*t*(13) = 5	*t*(12) = 5.8	*t*(14) = 8.2	*t*(12) = 0.6
	*p* < 0.001	*p* < 0.001	*p* < 0.001	*p* = 0.528
	*α*_adjusted_ = 0.004	*α*_adjusted_ = 0.004	*α*_adjusted_ = 0.003	*α*_adjusted_ = 0.004
Elite (highest outdoor redpoint 5.12b+)				
Before	*M* = 106 lbs	*M* = 99.5 lbs	*M* = 84 s	*M* = 17.4 s
	SD = 16.1	SD = 16.5	SD = 13.8	SD = 2.4
After	*M* = 90.3 lbs	*M* = 84.1 lbs	*M* = 15.5 s	*M* = 15.7 s
	SD = 18.6	SD = 20.9	SD = 12.9	SD = 1.8
Change	Δ = 15.7 lbs, −14.8%	Δ = 15.4 lbs*, −15.5%	Δ = 68.5 s*, −81.5%	Δ = 1.7 s, −9.8%
	*t*(7) = 3.5	*t*(6) = 8.1	*t*(6) = 9.5	*t*(5) = 1.8
	*p* = 0.011	*p* < 0.001	*p* < 0.001	*p* = 0.127
	*α*_adjusted_ = 0.007	*α*_adjusted_ = 0.007	*α*_adjusted_ = 0.007	*α*_adjusted_ = 0.008

* Indicates *p* < *α*_adjusted_.

Right hand grip strength decreased by 0.9 lbs per 1,000 ft climbed and 3.1 lbs per 100 routes climbed. Left hand grip strength decreased by 1.5 lbs per 1,000 ft climbed and 5 lbs per 100 routes climbed. Hang time decreased by 5.4 s per 1,000 ft climbed and 21 s per 100 routes climbed. Knot tying time increased by 1.7 s per 1,000 ft climbed and 6.5 s per 100 routes climbed. Fitted regression models are summarized in Table 2.

**Table 2:** Fitted regression models.

	Right hand grip strength	Left hand grip strength	Hang time	Knot tying time
Vertical height	*Ŷ* = 11.34 + 0.0008834*(ft)	*Ŷ* = 8.1453 + 0.001469*(ft)	*Ŷ* = 32.3137 + 0.005373*(ft)	*Ŷ* = 11.3479 − 0.001687*(ft)
	*R*^2 ^= 0.0069	*R*^2 ^= 0.03	*R*^2 ^= 0.12	*R*^2 ^= 0.026
	*F*(1,34) = 0.24	*F*(1,33) = 1.03	*F*(1,33) = 4.4	*F*(1,32) = 0.85
	*p* = 0.629	*p* = 0.318	*p* = 0.044	*p* = 0.363
	*β* = 0.0009	*β* = 0.0015	*β* = 0.0054	*β* = −0.0017
Route number	*Ŷ* = 11.8749 + 0.03083*(#)	*Ŷ* = 9.1435 + 0.05029*(#)	*Ŷ* = 33.1412 + 0.211*(#)	*Ŷ* = 10.9732 − 0.06511*(#)
	*R*^2 ^= 0.0052	*R*^2 ^= 0.022	*R*^2 ^= 0.11	*R*^2 ^= 0.024
	*F*(1,34) = 0.18	*F*(1,33) = 0.73	*F*(1,33) = 4.12	*F*(1,32) = 0.77
	*p* = 0.677	*p* = 0.399	*p* = 0.05	*p* = 0.386
	*β* = 0.031	*β* = 0.05	*β* = 0.21	*β* = −0.065

* Indicates multiplication.

^#^ Indicates number of routes.

## Discussion

### Generalizability

The competitors in the 24 h of Horseshoe Hell competition are generally recreational climbers that climb in their free time, without significant income from brand sponsorships, and who do not climb as their primary profession. Therefore, we believe that the data from the sampled participants can be applied broadly to recreational climbers worldwide.

Additionally, as the competition take place outdoors, on real rock faces, and requires partner teams to clip bolts, place gear, clean gear, and swap belays, we feel that this most accurately parallels multipitch outdoor climbing. Given that this competition was timed at 24 h, requiring teams to manage their hydration, food consumption, rest, waiting in line for routes, and toileting along with achieving their climbing goals, the results from this study are most relevant to climbers planning to spend half to a full day on a climbing objective.

An example of a popular climbing route where this could be applicable is the route Epinephrine, a 13-pitch, 1,600 ft, 5.9 that is often timed car-to-car ranging from 5 h 30 min to 20 h 30 min with an average time of 12 h 46 min for the 45 parties that ticked the climb in 2022 on Mountain Project, a community climbing forum (25). In 2022, 12 parties bailed off the route due to adverse conditions while 11 reported an “epic”, which is defined as “when a climb turns into an ordeal, often taking much longer than anticipated by being affected by adverse conditions or unexpected difficulties” (26). While none reported injury or requiring rescue, climbers who become fatigued are more prone to experiencing a climbing accident.

Aspiring climbers training for this route could measure their baseline grip strength and hang time, go climb for several hours, and then re-measure their grip strength and hang time once fatigued and compare the change they experience with the change from the competitors in this study to predict when they would reach the level of fatigue equivalent to 24 h of climbing, and therefore predict whether they would be likely to epic or require rescue.

### Results interpretation

This study found that competitors' grip strength decreased due to fatigue from repeated use of forearm, hand, and finger musculature after 24 h of climbing. When divided into subgroups based on division, the decrease in hang time remained significant for all groups. Grip strength was significantly decreased in all but the Recreational subgroup. This is likely because recreational climbers have been shown to have similar handgrip strength to physically active non-climbers (27). It is worth noting that while the mean grip strengths of the Intermediate climbers were greater than those of the Advanced group, the Advanced group had longer hang times, disproving the common misconception that “stronger” climbers have better grip strength, as even non-climbers have been found to have similar grip strength as elite climbers (28).

The results from this study add to the pool of knowledge on handgrip strength change in rock climbers, which has been shown to decrease 22% in 5.12a climbers climbing until a fall (29) and 22.1%–23% in 5.9 climbers climbing for 30 min (21). We found that a 1.9%–9.2% decrease in 5.9 climbers, a 16.2%–20.1% decrease in 5.10d climbers, 14.5%–14.6% decrease in 5.12a climbers, and a 14.8%–15.5% decrease in 5.12b+ climbers over a 24-hour period.

Our study also looked at how the number of vertical feet climbed and the number of routes climbed could predict changes in grip strength, hang time, and knot-tying. Although these predictors did not reach significance when addressing grip strength or knot-tying times, we were able to predict with significance how vertical feet could affect hang time. The number of routes climbed did not predict any change in measurements, which indicates that the time spent climbing contributes more to fatigue than the number of iterations of changeovers from belay to climb and climb to belay.

Watts et al. has previously demonstrated that max time for repeated hangs plateaued with 1–3 min rest intervals, with a mean hang time of 36.3–40.7 s over 8 tries within <30 mins. In our study, mean hang time was 38.1 s after 24 h climbing, which is similar to the values they found. However, participants in our study had higher mean hang times before the competition (76.8 s) which may reflect the endurance competitors had built up in preparation for the event lasting multiple hours.

Hang time is a useful proxy for measuring climber endurance (30), especially when considering longer objectives for climbers, such as multi-pitch routes or big-wall climbing. A climber's self-selected speed of climbing is thought to be a balance between time doing isometric work and the avoidance of early muscle fatigue (31). Hangboard exercises have been proven to improve grip endurance after 4–8 weeks of training (32) and are a popular training technique to delay the onset of the dreaded forearm pump that ails climbers who become fatigued.

Our prediction model may be useful for climbers preparing for large outdoor objectives. By using their current maximum hang time as a data point for their present level of climbing fitness, climbers may utilize the predictors in this study to estimate their change in hang time after their desired objective and target their training to decrease this difference. Climbers may also aim for a certain hang time objective during their training period to maximize chances of a successful ascent and thus avoid unplanned overnight bivouacs or emergency calls for rescue. Climbers may also use the prediction model to compare different objectives and expected changes in their performance as a marker of fatigue.

### Limitations

There are several limitations that we encountered during our study. While we measured right and left hand grip strengths, we did not indicate which laterality was the climber's dominant hand, which would have been an interesting additional point of investigation. Climbers also gave varying degrees of effort during their pre-test hang times. While most competitors gave their maximal efforts during the pre- and post-test hang times, a handful of them indicated that they did not want to tire themselves out prior to the start of the competition for fear of overexertion, or “pumping themselves out,” and thus let go before what would otherwise have been their maximal hang time. Additionally, we did not measure climbers' height or weight to calculate a strength to body mass ratio, which has been shown to determine performance in indoor World Cub sport climbers (33).

This was an observational study, so we did not specify which routes each climber chose in the course of the competition. Climbers competing in the Elite category could select to climb the same number and difficulty of routes as someone competing in the Recreational category, and therefore may demonstrate a smaller change in their grip strength, hanging endurance, and knot tying speed by the end of the competition.

We were unable to standardize the exact times for when each measurement was taken after the competition. Thus, some competitors were measured directly after their finish, while others were measured up to an hour after the official finish. The order in which we took the three measurements also varied between climbers. Lastly, we were also unable to control for any possible effects of drugs used before or after the event.

This study was undertaken over the course of 24 h of consecutive competition. Of the 54 original participants, 18 did not present for repeat measurement immediately after the competition and were lost to follow-up. Additionally, some measurements were not recorded or not legible for interpretation. Therefore, the data presented in this study is limited by our small sample size.

### Future avenues of study

Repeating this study on shorter intervals of continuous climbing can provide insight into the level of fatigue expected after shorter climbing objectives. Replicating this study using a standard climbing harness and hangboard with a fixed grip width would better standardize the results and can also help recreational climbers repeat these measurements at home to assess their own fatigue after training sessions or unexpected days out.

## Data Availability

The original contributions presented in the study are included in the article, further inquiries can be directed to the corresponding author.
